# A comparative analysis of library prep approaches for sequencing low input translatome samples

**DOI:** 10.1186/s12864-018-5066-2

**Published:** 2018-09-21

**Authors:** Yang Song, Beatrice Milon, Sandra Ott, Xuechu Zhao, Lisa Sadzewicz, Amol Shetty, Erich T. Boger, Luke J. Tallon, Robert J. Morell, Anup Mahurkar, Ronna Hertzano

**Affiliations:** 1 Institute for Genome Sciences, University of Maryland School of Medicine, Baltimore, MD 21201 USA; 2 Department of Otorhinolaryngology-Head & Neck Surgery, University of Maryland School of Medicine, Baltimore, MD 21201 USA; 3 Genomics and Computational Biology Core, National Institute on Deafness and Other Communication Disorders, Bethesda, MD 20892 USA; 4 Department of Anatomy and Neurobiology, University of Maryland School of Medicine, Baltimore, MD 21201 USA

**Keywords:** RiboTag, Library preparation kits, Low-input RNA-seq, RNA-seq, Coverage bias

## Abstract

**Background:**

Cell type-specific ribosome-pulldown has become an increasingly popular method for analysis of gene expression. It allows for expression analysis from intact tissues and monitoring of protein synthesis in vivo. However, while its utility has been assessed, technical aspects related to sequencing of these samples, often starting with a smaller amount of RNA, have not been reported. In this study, we evaluated the performance of five library prep protocols for ribosome-associated mRNAs when only 250 pg-4 ng of total RNA are used.

**Results:**

We obtained total and RiboTag-IP RNA, in three biological replicates. We compared 5 methods of library preparation for Illumina Next Generation sequencing: NuGEN Ovation RNA-Seq system V2 Kit, TaKaRa SMARTer Stranded Total RNA-Seq Kit, TaKaRa SMART-Seq v4 Ultra Low Input RNA Kit, Illumina TruSeq RNA Library Prep Kit v2 and NEBNext® Ultra™ Directional RNA Library Prep Kit using slightly modified protocols each with 4 ng of total RNA. An additional set of samples was processed using the TruSeq kit with 70 ng, as a ‘gold standard’ control and the SMART-Seq v4 with 250 pg of total RNA. TruSeq-processed samples had the best metrics overall, with similar results for the 4 ng and 70 ng samples. The results of the SMART-Seq v4 processed samples were similar to TruSeq (Spearman correlation > 0.8) despite using lower amount of input RNA. All RiboTag-IP samples had an increase in the intronic reads compared with the corresponding whole tissue, suggesting that the IP captures some immature mRNAs. The SMARTer-processed samples had a higher representation of ribosomal and non-coding RNAs leading to lower representation of protein coding mRNA. The enrichment or depletion of IP samples compared to corresponding input RNA was similar across all kits except for SMARTer kit.

**Conclusion:**

RiboTag-seq can be performed successfully with as little as 250 pg of total RNA when using the SMART-Seq v4 kit and 4 ng when using the modified protocols of other library preparation kits. The SMART-Seq v4 and TruSeq kits resulted in the highest quality libraries. RiboTag IP RNA contains some immature transcripts.

**Electronic supplementary material:**

The online version of this article (10.1186/s12864-018-5066-2) contains supplementary material, which is available to authorized users.

## Background

Considerable scientific effort has been dedicated to understanding cell type-specific expression profiles from complex tissues, such as brain, liver, pancreas, testes, eye or ear [1–7]. To overcome the issue of cellular heterogeneity within complex tissues, two methods have been traditionally used in mice: Laser-Capture Microdissection (LCM) [8, 9] and Fluorescence Activated Cell Sorting (FACS) [10, 11]. However, LCM is a laborious and time-consuming procedure with low yield of mRNA; and FACS requires tissue dissociation – which may lead to changes in gene expression – and requires dedicated equipment [12]. More recently, single cell RNA-seq has been introduced. However, this technique too requires tissue dissociation and is currently limited by the number of genes detected per sequenced cell [13, 14]. To overcome these limitations, Translating Ribosome Affinity Purification (TRAP) [15] and RiboTag [16] have been recently developed to study cell type-specific transcriptome profiles. Both methods rely on immunoprecipitation of ribosome-attached RNA (also named ‘translatome’) by cell type-specific molecular targeting of the ribosomal proteins, often in a Cre-lox based system [15–17]. These methods have the advantage of not requiring tissue dissociation, thus allowing for cell type-specific translatome analysis from intact tissues.

While ribosome-attached RNA sequencing for expression analysis has been validated from a biological standpoint [18, 19], the technical aspects of its library construction and sequencing have not been studied. In instances where small complex tissues are studied, the amount of starting material after immunoprecipitation may be limited (e.g., less than 5 ng). When starting from low amounts of RNA, additional cycles of amplification using PCR are performed after adapter ligation to amplify the cDNA to generate enough material for sequencing. Multiple commercial kits are available in the market to build cDNA libraries from samples with low amounts of RNA, including kits from NuGEN, New England Biolabs (NEB), Illumina and TaKaRa. Standard protocols for library construction are commonly designed to start with more than 100 ng of total RNA [20, 21] and only a few studies have been conducted to compare the performance of library preparation kits using less than 5 ng of total RNA as their starting amount [22, 23]. In this study, we selected four of the commonly used library preparation kits that are also suitable for lower-input samples for comparison. We modified the standard protocols for NEB and Illumina library preparation kits to enable them to work with smaller amounts of RNA than the recommended amounts down to as little as 4 ng of total RNA. We included one kit, SMART-Seq v4, that was designed for single cell RNA-seq and tested it with 4 ng and 250 pg of total RNA. We evaluated the performance of the different kits based on duplication rate, percentage of intronic and exonic regions being detected, the evenness of coverage of transcripts and ribosomal RNA read-count in comparison to total reads. We also compared the reproducibility of the enrichment or depletion effect based on ribotag-translatome profile for the first time.

## Methods

### Animals

The *Gfi1*-Cre knock-in mice generated by Dr. Lin Gan (University of Rochester) were kindly provided by Dr. Jian Zuo of the Developmental Neurobiology Department at St. Jude Children’s Research Hospital. RiboTag mice generated by Dr. Paul S Amieux (University of Washington) were kindly provided by Dr. Mary-Kay Lobo of the Department of Neurobiology and Anatomy at University of Maryland Baltimore. B6.Cg-Gt(ROSA)26Sor^tm14(CAG-tdTomato)Hze^/J mice (also referred to as Ai14) were purchased from the Jackson Laboratory (stock #007914, Bar Harbor, ME). Experimental animals for RNA-seq, *Gfi1*^*Cre/+*^*;RiboTag*^*HA/HA*^, were obtained by crossing RiboTag mice with Gfi1-Cre mice. Animals for immunostaining, *Gfi1*^*Cre/+*^*;Ai14*, were obtained by crossing Gfi1-Cre mice with Ai14 mice [24]. All procedures involving animals were carried out in accordance with the National Institutes of Health Guide for the Care and Use of Laboratory Animals and have been approved by the Institutional Animal Care and Use Committee at the University of Maryland, Baltimore (protocol numbers 1015003 and 0915006).

### Ribosome immunoprecipitation and RNA extraction

Three 30-day old *Gfi1*^*Cre/+*^;*RiboTag*^*HA/HA*^ mice were euthanized by CO_2_ asphyxiation followed by cervical dislocation. Livers were harvested and immediately frozen on dry ice. Equal amounts of liver were used for input RNA extractions (RNeasy Plus Micro kit, QIAGEN USA, Germantown, MD, USA) or futher processed for ribosome immunoprecipitation (5 μg of purified anti-HA.11, BioLegend, San Diego, CA, USA) followed by RNA extraction as previously decribed in Sanz et al., 2009 [16]. The RNeasy Plus Micro kit is optimized for the removal of genomic DNA through a combination of high salt buffer and the gDNA Eliminator spin column. Quality of the RNA was assessed on an Agilent Technologies Bioanalyzer 2100 RNA pico chip as per the manufacturer’s instructions (Agilent Technologies, Palo Alto, CA, USA). All samples had a RIN score of 10 and no evidence of DNA contamination in the form of a high molecular weight DNA band. All RNA was equally aliquoted to test for the performance of five commercial kits and seven protocols.

### Real-time RT-PCR

Efficiency of the ribosome immunoprecipitation was assessed by reverse transcription followed by real time PCR. One nanogram of total RNA from the input and the IP samples was used for reverse transcription using the Maxima First Strand cDNA Synthesis Kit for RT-qPCR (Thermo Fisher Scientific, Waltham, MA, USA). The real time PCR was performed on an Applied Biosystems® StepOnePlus™ Real-Time PCR System with the Maxima SYBR Green/ROX qPCR Master Mix (Thermo Fisher Scientific, Waltham, MA, USA) and the following primers: *Gapdh*-Fw 5’-GGAGAAACCTGCCAAGTATGA-3′; *Gapdh*-Rv 5′- TCCTCAGTGTAGCCCAAGA-3′; *Gfi1*-Fw 5′- AATGCAGCAAGGTGTTCTC-3′; *Gfi1*-Rv 5′- CTTACAGTCAAAGCTGCGT-3′.

### Immunostaining

Progeny from a cross between *Gfi1*^*Cre/+*^ mice and TdTomato reporter mice Ai14 were euthanized at P1 and their livers were harvested. Following fixation in 4% paraformaldehyde overnight at 4 °C, livers were cryoprotected through incubation in PBS with increasing amount of sucrose before being embedded in O.C.T. compound (Scigen, Gardena, CA, USA). Ten μm cryosections were permeabilized with PBS supplemented with 0.2% Tween-20 for 1 h at room temperature and incubated with Alexa Fluor™ 488 Phalloidin (1/800, Thermo Fisher Scientific, Waltham, MA, USA) and DAPI (1/20,000, Thermo Fisher Scientific, Waltham, MA, USA). Samples were mounted with ProLong Gold antifade reagent (Thermo Fisher Scientific, Waltham, MA, USA). Images were acquired using a Nikon Eclipse E600 microscope (Nikon, Tokyo, Japan) equipped with a Lumenera Infinity 3 camera (Lumenera, Ottawa, ON).

### RNA-Seq library construction

Below are the experimental methods for RNA-Seq library construction. We followed the manufacturer’s instructions with minor modifications, as noted below. The shearing approach was not altered and remains different between kits.

#### Ovation® RNA-Seq system V2 combined with TruSeq RNA library prep kit v2

We performed a hybrid library preparation by using Ovation® RNA-Seq System V2 (NuGEN, San Carlos, CA, USA) to synthesize cDNA and the TruSeq RNA Library Preparation Kit v2 to construct the sequencing library (Illumina, San Diego, CA, USA), consistent with the NuGEN manufacturer protocol (See Additional file 1: Table S1). Briefly, 4 ng of total RNA or RiboTag IP RNA were used to synthesize cDNA following the NuGEN’s instructions. Subsequently, 200 ng of cDNA were sheared to an average size of 300 bp with a Covaris E220 Focused-Ultrasonicator (Covaris Inc., Woburn, MA, USA). Following the manufacturer protocol, the library was prepared from the sheared cDNA using the Illumina TruSeq RNA Library Prep Kit with 8 cycles of PCR.

#### SMARTer® stranded total RNA-Seq kit-Pico input mammalian

Four nanograms of RNA were used as input material and libraries were prepared by following the SMARTer Stranded Total RNA-Seq kit-Pico Input Mammalian user manual (Takara Bio USA, Mountain View, CA, USA). In brief, samples were fragmented at 94 °C for 4 min prior to first-strand synthesis. Illumina adaptors and indexes were added to single-stranded cDNA via 5 cycles of PCR. Libraries were hybridized to R-probes for fragments originating from ribosomal RNA to be cleaved by ZapR. The resulting ribo-depleted library fragments were amplified with 15 cycles of PCR.

#### SMART-Seq® v4 ultra® low input RNA kit for sequencing

Two types of libraries were prepared by using 4 ng or 250 pg RNA from each sample. Libraries were prepared by following the SMART-Seq v4 Ultra Low Input RNA Kit (Takara Bio USA, Mountain View, CA, USA) user manual. The cDNA was amplified with 11 cycles of PCR. Nextera XT kit (Nextera XT DNA Library Preparation Kit (Illumina, San Diego, CA) was used to make cDNA libraries suitable for Illumina sequencing.

#### TruSeq RNA library prep kit v2

Two types of libraries were prepared by using 70 ng or 4 ng RNA from each sample. The 70 ng libraries were built using TruSeq RNA Library Prep Kit v2 (Illumina, San Diego, CA, USA) according to the manufacturer protocol. Size selection was performed using SPRIselect beads (Beckman Coulter, Indianapolis, IN, USA) and in-house calibration values (first round selection to select the upper or right limit of the distribution), salt unit equals to 0.427 and second round selection to select the lower or left limit of the distribution, salt unit = 0.455). The cDNA was amplified with 19 cycles of PCR. Libraries were prepared using 4 ng of RNA with modifications to the standard protocol by reducing the end-repair reaction to 1/2 the recommended amounts of enzyme mix and sample volume. In addition A-tail ligation followed the standard protocol without the use of internal control mixes. Due to the low input amount, no size selection was applied to the 4 ng libraries. The cDNA was amplified with 22 cycles of PCR. Libraries prepared using 70 ng of RNA were prepared following the standard protocol and cDNA was amplified with 14 cycles as suggested by manufacture protocol.

#### NEBNext® ultra™ directional RNA library prep kit for Illumina

Four nanograms of total RNA were used for NEBNext® Ultra™ Directional RNA Library Prep Kit (New England Biolabs, Ipswich, MA, USA). Poly-A selection and cDNA synthesis were performed according to NEB protocol. The adaptors were diluted with a 1:30 ratio instead of the recommended 1:10 ratio. Size selection was performed using SPRIselect beads. (Beckman Coulter, Indianapolis, IN, USA) with in-house calibration values. The cDNA was amplified with 22 cycles of PCR.

### Sequencing

Samples prepared by TruSeq, NEB, NuGEN and SMARTer were sequenced at the Institute for Genome Sciences (IGS) Genomics Resource Center (Baltimore, MD) on a HiSeq 4000 using 75 base read lengths in paired-end mode. Samples prepared by SMART-Seq v4 were sequenced by the Genomics and Computational Biology Core (GCBC) at the National Institute on Deafness and Other Communication Disorders (NIDCD/NIH) on a HiSeq 1500 using a read length of 126 bases in paired-end mode.

### RNA-Seq analyses

The Illumina adapters used during the library construction were removed from the reads using Trimmomatic [1]. In order to reduce the impact of lower quality reads on the alignment, all reads were trimmed to 60 bp using the FASTX Toolkit v-0.0.13 [25] resulting in a Phred-Quality-Score greater than 30. The reads generated for each RNA sample were analyzed and compared using an Ergatis-based RNA-Seq analysis pipeline [26] where sequencing reads were aligned and annotated to the UCSC mouse reference genome (mm10, GRCm38.84) from Ensembl (http://www.ensembl.org) using TopHat v-2.0.8 [27] (maximum number of mismatches   =   2; segment length   =   30; maximum multi-hits per read   =  25; maximum intron length   =   50,000) and the number of reads that aligned to the predicted coding regions were determined using HTSeq [28]. Bedtools (v-2.7.1) [29] was used to count the reads mapping to exons according to Ensembl gene annotations (March 2016, Mus_musculus.GRCm38.84, with 47,729 genes annotated). Read counts per million mapped reads values (CPM) [28] or reads per kilobase of transcripts per million mapped reads (RPKM) [30] were calculated and used for downstream analyses. 5′-3′ exonic coverage was calculated using CollectRnaSeqMetrics component of picard-tools (v-1.60, https://broadinstitute.github.io/picard/), and duplication rate was calculated using EstimateLibraryComplexity of Picard-tools.

### Statistical analysis

All plots were generated using R (v-3.2.4), including the following R packages ggplot2, ComplexHeatmap for producing bar plots or heat maps, and limma to generate Venn diagrams. The difference among groups in boxplots was evaluated based on the overlapping of the notch region [31]. The notch is defined as median m ± 1.58IQR/√n [31]. The significance test is evaluated using a non-parametric Wilcoxon test with *p* < 0.05.

### Accession number

All of the processed gene expression data from this study have been submitted to the NCBI Gene Expression Omnibus (GEO) under accession number GSE104213.

## Results

### Sample preparation for sequencing

In order to evaluate the efficiency of different library preparation kits with low amounts of RNA obtained after ribosome immunoprecipitation, we crossed RiboTag mice with *Gfi1*-Cre mice to obtain progeny that expressed HA-tagged ribosomes in cells with *Gfi1* expression. We obtained RNA from liver because it is a tissue that, at least during embryogenesis, expresses *Gfi1*, thus allowing for early induced recombination in a subset of the liver cells for permanent expression of a reporter gene [32] (Additional file 2: Figure S1a). Additionally, the size of the liver would provide enough material to test five different kits with varying amounts of starting RNA from individually processed samples. Livers were processed for HA-tagged ribosome immunoprecipitation (IP) followed by RNA extraction as previously described [16]. Prior to sequencing, the efficacy of the IP was confirmed by comparing the level of *Gfi1* transcripts in the input and IP samples using real time RT-PCR (Additional file 2: Figure S1b). The profiles generated by the five different commercial library preparition kits, from four different manufacturers, were compared in this study (See Additional file 1: Table S1). NEBNext® Ultra™ Directional RNA Library Prep Kit for Illumina (NEB) with 4 ng of RNA,NuGEN Ovation® RNA-Seq System V2 with 4 ng of RNA (NuGEN) with 4 ng of RNA, TaKaRa SMARTer® Stranded Total RNA-Seq Kit-Pico Input Mammalian with 4 ng of RNA (SMARTer), TaKaRa SMART-Seq® v4 Ultra® Low Input RNA Kit for Sequencing with 4 ng and 0.25 ng of RNA (SMARTseq4 and SMARTseq0.25) and Illumina TruSeq RNA Library Prep Kit v2 with 4 ng and 70 ng of RNA (TruSeq4 and TruSeq70). Of these kits only the SMARTer kit produced strand specific libraries and we therefore did not analyze the data for strandness.

### Comparison of mapping efficiency and duplication rate

**Fig. 1:**
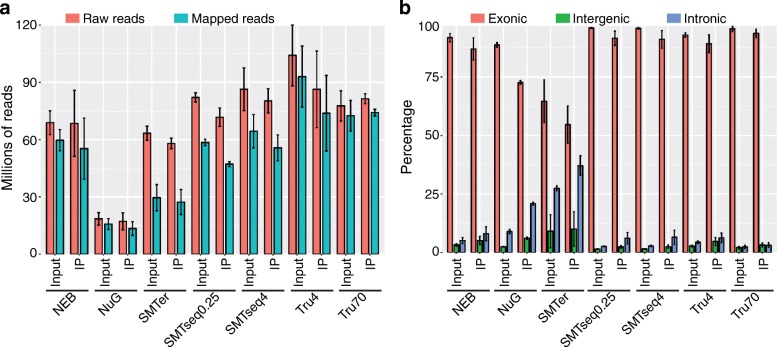
Descriptive characteristics of raw and mapped reads. **a** Total number of raw reads and number of reads mapped to the mouse genome (mm10, GRCm38.84). **b** Percentage of reads mapped to exonic, intronic and intergenic regions. NEB: NEBNext® Ultra™, NuG: NuGEN Ovation®, SMTer: SMARTer® Stranded; Tru4: TruSeq using 4 ng of RNA; Tru70: TruSeq using 70 ng of RNA. SMTseq4: SMART-Seq® v4 using 4 ng of RNA; SMTseq0.25: SMART-Seq® v4 using 250 pg of RNA

The number of reads varied widely among samples being prepared by different library preparation kits. Input RNA samples generated 14.7 to 122 million pair-end reads (2 × 60 bp) and IP RNA samples generated 12 to 108 million pair-end reads (2 × 60 bp). Overall, fewer raw/mapped reads were generated when using the NuGEN kit. Of the raw reads, 12.5 to 111 million reads mapped to mouse genome for input RNA samples while 9.2 to 94.6 million reads mapped for IP RNA samples (Fig. 1a).

In order to evaluate the expression profile composition and library complexity, we assessed the duplication rate of the read pairs (Table 1) as lower duplication rates usually indicate a higher complexity of the sample and better representation of RNA present in a sample [20]. In this study, duplication rate ranged from 26 to 99% (Table 1). However, the duplication we observed was not well correlated with the numbers of PCR cycles and was more dependent on the library prep kit. For instance, while NEB and TruSeq4 samples both had the highest number of PCR cycles (22), their duplication rates differed (Table 1). Indeed, NEB-input samples had the highest duplication rate of 99% with the overall largest number of reads duplicated more than 200 times while the TruSeq4 samples had a duplication rate of 72% with a substantially smaller number of reads with greater than 200 duplications (Table 1 and Additional file 3: Figure S2).

**Table 1:** Duplication rate of libraries prepared by different kits

Kit	Sample type	PCR cycles for lib prep	Percent_Duplication (%)			
			replicate			avg
			1	2	3	
NEB^a^	Input	22	97	99	97	97
	IP		99	99	99	99
NuG/Tru^b^	Input	8	52	53	52	53
	IP		36	31	26	31
SMTer^c^	Input	15	83	86	85	85
	IP		86	83	68	79
Tru4^d^	Input	22	67	77	71	72
	IP		85	82	99	89
Tru70^e^	Input	19	90	94	92	92
	IP		98	97	96	97
SMTseq4^f^	Input	11	40	38	40	40
	IP		60	49	48	52
SMTseq0.25^f^	Input	11	37	37	41	38
	IP		59	50	47	52

^a^NEBNext® Ultra™ Directional RNA Library Prep Kit for Illumina

^b^Nugen Ovation® RNAseq System v2

^c^SMARTer® Stranded Total RNA-Seq Kit-Pico Input Mammalian User

^d^TruSeq® RNA sample preparation v2,4 ng

^e^TruSeq® RNA sample preparation v2,70 ng

^f^SMART-Seq v4 Ultra Low Input RNA Kit

### Detection of exonic, intergenic and intronic regions

Among the mapped reads, SMARTer samples showed the lowest alignment to exonic regions. The percentage of reads aligned to exonic regions was greater than 85% in samples prepared with NEB, TruSeq and SMARTseq library kits and less than 70% in samples prepared with the SMARTer and NuGEN kits (Fig. 1b). As expected, the overall percentage of reads aligning to intronic regions detected in input samples was less than 10% for most samples, except for samples prepared by the SMARTer kit, where more than 20% of the reads align to intronic regions. IP samples had roughly twice as many reads aligning to intronic regions, or 10% more intronic reads compared with the corresponding input RNA samples, which may suggest that the IP captures some immature mRNAs. In particular, the percentage of intronic reads from the SMARTer samples increased from 22% for the input RNA to 41% for the IP RNA. The percentage of intronic reads for the NuGEN samples ranged from 8% for the input RNA and 22% for the IP RNA.

### Number of genes being detected as expressed

**Fig. 2:**
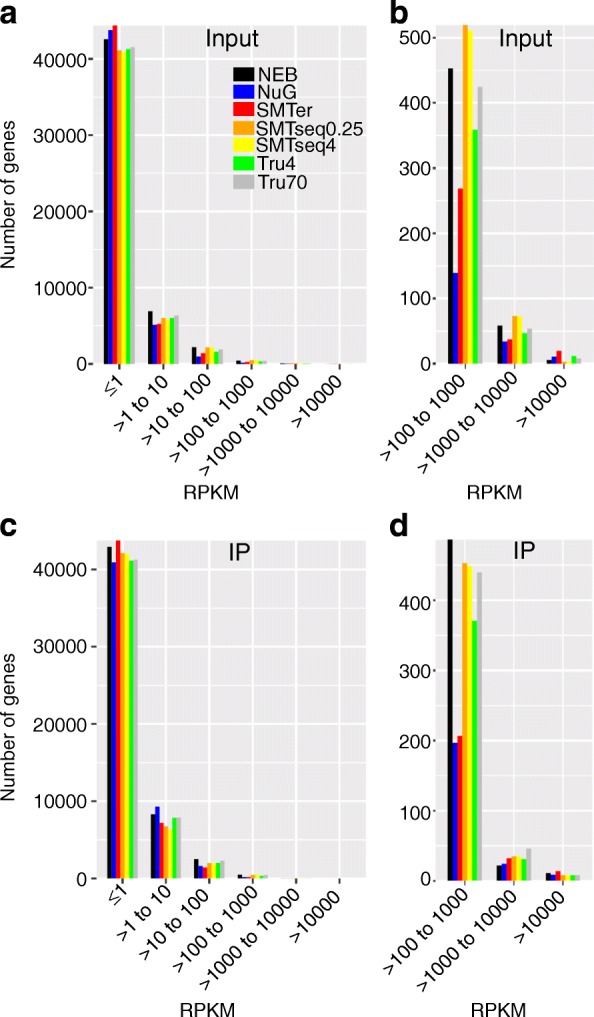
Detected transcripts binned by RPKM. There were 33,399 transcripts detected in at least one input sample, 35,395 transcripts detected in at least one IP sample and all of these genes were used for the binning. Y-axis: number of transcripts detected in at least one replicate. **a** and **c** Number of genes binned by RPKM as detected by the different kits in the input and IP, respectively. **b** and **d** These plots are subsets of (**a**) and (**c**) focusing only on highly expressed transcripts (RPKM> 100)

Because of the differences in sequencing efficiency and library complexity, we examined the number of features detected in samples prepared with each library kit. After removing ambiguous reads or reads mapped to multiple features using HTSeq, we detected between 10,184 to 21,161 genes where the CPM values were greater than zero (Table 2). The corresponding average raw read counts ranged from 0.76 to 37 million reads. Fewer features were detected in SMARTer and NEB samples. All of the annotated genes (47,729) were binned into 6 groups (RPKM≤1, 1 < RPKM≤10, 10 < RPKM≤100, 100 < RPKM≤1000, 1000 < RPKM≤10,000 and RPKM> 10,000. Fig. 2). SMARTer, NuGEN, and NEB samples had more genes that were entirely missed or had low expression levels (RPKM≤1) in comparison to the other kits in the input samples (Fig. 2a). The SMARTer and NEB samples had more genes with a lower expression levels (RPKM≤1) also in the IP (Fig. 2c). The number of genes within RPKM range (100–10,000) was relatively low in SMARTer and NuGEN samples (Fig. 2b, d). Conversely, SMARTer samples contained more highly expressed genes (RPKM> 10,000) than others samples, but the majority of these were rRNA genes or genes encoding for hypothetical proteins (Fig. 2b, d and See Additional file 1: Table S2).

**Table 2:** Number of features with CPM > 0

Kit	Sample type	Number of features with coverage (CPM > 0)				Number of reads of all expressed features (CPM > 0)			
		Replicate			avg	Replicate			avg
		1	2	3		1	2	3	
NEB	Input	13,543	12,464	11,419	12,475	22,373,163	26,292,017	22,305,929	23,657,036
	IP	11,059	12,165	14,331	12,518	16,668,355	27,323,849	15,122,257	19,704,820
NuG	Input	16,946	16,458	15,624	16,343	5,529,417	7,615,866	7,482,819	6,876,034
	IP	17,715	17,841	18,198	17,918	3,223,153	5,319,712	5,141,329	4,561,398
SMTer	Input	12,281	10,184	10,929	11,131	759,221	762,562	750,159	757,314
	IP	12,422	11,168	15,684	13,091	701,059	645,414	464,199	603,557
Tru4	Input	19,906	19,863	19,631	19,800	35,368,451	45,466,334	31,299,550	37,378,112
	IP	17,467	18,899	21,161	19,176	36,785,680	28,571,125	18,031,264	27,796,023
Tru70	Input	15,957	15,577	16,082	15,872	32,103,296	33,797,108	25,797,283	30,565,896
	IP	14,430	15,213	15,680	15,108	31,150,532	29,799,544	27,902,766	29,617,614
SMTseq4	Input	19,111	19,287	19,630	19,343	23,573,272	24,615,731	30,217,161	26,135,388
	IP	19,835	20,551	20,488	20,291	14,407,184	19,577,709	17,262,557	17,082,483
SMTseq0.25	Input	16,834	16,742	16,117	16,564	23,815,261	24,347,672	22,951,893	23,704,942
	IP	16,827	16,870	16,527	16,741	13,971,566	15,047,219	14,691,099	14,569,961

**Fig. 3:**
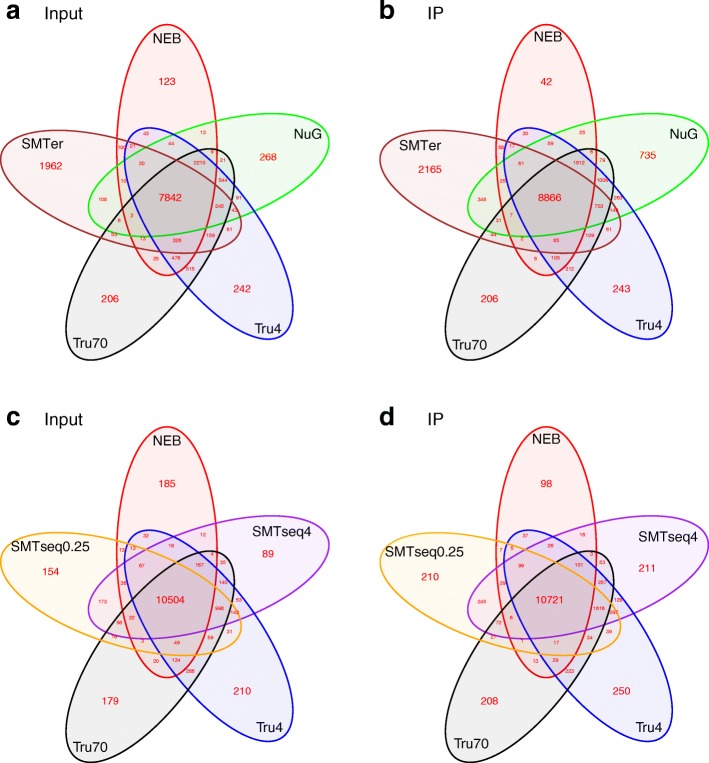
Venn diagrams of identified features in the different libraries. The features with CPM ≥ 1 in at least one out of 3 replicates were used to generate these plots. **a** and **c** represent input samples and **b** and **d** represent IP samples. Most transcripts were detected by all kits tested. However, a higher rate of agreement is seen between the NEB, TruSeq and SMART-Seq prepared samples. NEB: NEBNext® Ultra™, NuG: NuGEN Ovation®, SMTer: SMARTer® Stranded; Tru4: TruSeq using 4 ng of RNA; Tru70: TruSeq using 70 ng of RNA. SMTseq4: SMART-Seq® v4 using 4 ng of RNA; SMTseq0.25: SMART-Seq® v4 using 250 pg of RNA

In order to compare the similarity of expression profiles of the different kits, we compared genes with at least 1 CPM in at least one replicate across all the samples. More than 60% of the genes were co-detected by all kits (Fig. 3). The median CPM for shared genes across all samples was 28 for input samples **(**Fig. 3a, c**)** and 36 for IP samples **(**Fig. 3b, d**)**. Meanwhile, less than 10% of features were uniquely detected in NEB, NuGEN and TruSeq input samples, but over 20% of features were uniquely detected from the SMARTer samples. The median CPM of uniquely detected genes in SMARTer input samples was around 10, while the median for other kits was less than 3. A similar trend is observed in IP samples (Fig. 3b, d and See Additional file 1: Table S3).

We also grouped all of the detected features into ribosomal RNA, non-coding (ribosomal RNA, lincRNA, microRNA) and protein-coding groups. The average CPM of ribosomal RNA and non-coding gene groups were 2-fold higher in NuGEN and SMARTer samples than in other samples (Table 3). Conversely, the average CPMs for the protein-coding group were similar across most samples, except for SMARTer prepared samples (Table 4). By comparing IP samples with input samples, it is interesting that the CPMs of IP samples are relatively lower than input samples, except for NuGEN prepared samples.

**Table 3:** Descriptive statistic of non-coding detected features (CPM > =1)

Kit	Sample type	Total features of ribosomal RNA			Average CPM of ribosomal RNA			Total features of ribosomal RNA, lincRNA, microRNA			Average CPM of ribosomal RNA, lincRNA, microRNA		
		Replicate			Replicate			Replicate			Replicate		
		1	2	3	1	2	3	1	2	3	1	2	3
NEB	Input	3	3	3	73.87	67.33	72.15	233	186	140	46.01	29.22	28.34
	IP	3	3	4	14.24	6.15	7.80	128	157	268	78.05	28.33	34.25
NuG	Input	8	11	10	5533.70	5571.81	5603.65	552	488	415	272.73	274.17	276.54
	IP	13	11	17	1803.34	820.73	863.91	583	593	637	97.91	48.26	51.37
SMTer	Input	8	5	6	4282.62	7858.83	4641.15	440	359	377	247.75	429.65	261.04
	IP	12	6	17	4880.27	7906.14	3936.78	467	411	599	282.51	429.64	240.08
Tru4	Input	17	17	13	232.50	120.94	169.47	804	786	767	13.96	7.59	10.29
	IP	8	7	19	415.93	129.19	168.59	546	650	927	26.49	8.94	11.45
Tru70	Input	6	4	4	102.39	28.55	58.50	410	363	406	6.34	2.08	3.81
	IP	3	3	4	141.53	54.91	65.21	265	295	341	9.24	3.97	5.10
SMTseq4	Input	13	13	21	214.51	159.76	185.17	750	764	803	11.78	8.93	10.54
	IP	7	11	10	851.62	63.75	92.92	844	881	889	46.39	5.91	8.73
SMTseq0.25	Input	6	7	3	224.50	191.46	235.20	531	515	461	12.32	10.52	13.04
	IP	6	6	7	886.27	73.72	99.28	476	502	457	48.03	6.43	8.88

**Table 4:** Descriptive statistic of protein-coding detected features (CPM ≥ 1)

Kit	Sample type	Total features of protein-coding RNA			Average CPM of protein-coding RNA		
		Replicate			Replicate		
		1	2	3	1	2	3
NEB	Input	11,316	10,551	9789	36.51	37.77	37.83
	IP	8740	10,252	12,435	34.13	37.84	37.39
NuG	Input	10,921	10,828	10,656	19.61	19.50	19.32
	IP	12,970	13,142	13,318	32.65	36.35	36.12
SMTer	Input	11,136	9368	10,019	21.47	7.90	20.48
	IP	11,826	11,860	11,824	18.88	7.90	22.02
Tru4	Input	11,826	11,860	11,824	38.90	39.38	39.18
	IP	12,360	12,842	12,941	37.97	39.28	39.09
Tru70	Input	11,690	11,691	11,611	39.47	39.79	39.66
	IP	11,996	12,493	12,660	39.26	39.65	39.56
SMTseq4	Input	11,659	11,499	11,578	39.07	39.28	39.16
	IP	12,443	12,579	12,587	36.50	39.51	39.01
SMTseq0.25	Input	11,500	11,475	11,384	39.03	39.16	38.97
	IP	12,648	12,846	12,940	36.38	39.47	39.29

### Coverage uniformity relative to 5′ and 3′ ends

**Fig. 4:**
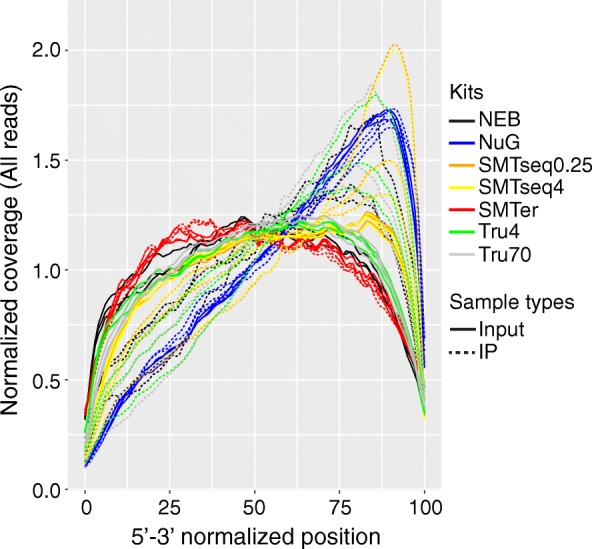
Distribution of normalized mean expression of the first (last) 100 bases of transcripts (in 5′- > 3′-orientation). X axis represents the 5′-3′ normalized position; Y axis represents normalized coverage. NEB: NEBNext® Ultra™, NuG: NuGEN Ovation®, SMTer: SMARTer® Stranded; Tru4: TruSeq using 4 ng of RNA; Tru70: TruSeq using 70 ng of RNA. SMTseq4: SMART-Seq® v4 using 4 ng of RNA; SMTseq0.25: SMART-Seq® v4 using 250 pg of RNA. Yellow and orange: SMTseq samples; Red: SMTer samples; Black: NEB samples; Blue: NuG samples; Green and grey: TruSeq samples. Solid: Input samples. Dotted: Ribo-IP samples

The evenness of transcript coverage was calculated by dividing the mean coverage of first (last) 100 bases (5′ or 3′) of transcripts divided by the mean coverage of all bases across the corresponding transcript (Fig. 4). The median was calculated and plotted for the 1000 most highly expressed transcripts. Most of the input RNA samples showed even coverage from 5′ to 3′ end, except for all NuGEN samples which had pronounced increase in coverage at the 3′ end. Additionally, consistently higher coverage at the 3′ end was observed among IP RNA samples, except for SMARTer samples with even coverage across 5′ and 3′ extremities.

### Similarity of expression profiles

**Fig. 5:**
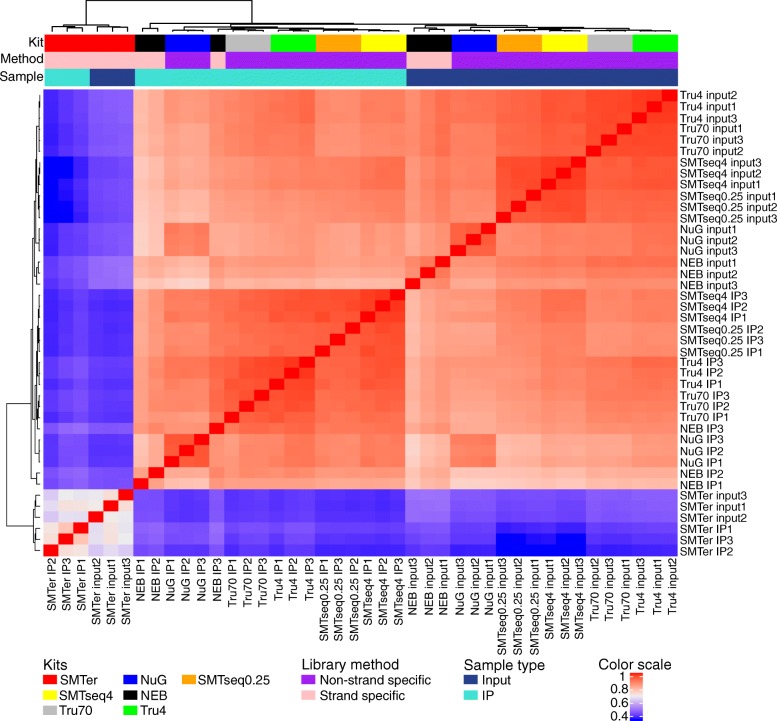
Hierarchical clustering of expression levels, based on the rank of the count of exon per million mapped reads (CPM). Dendrogram represents Spearman correlation coefficients between pairs of samples. NEB: NEBNext® Ultra™, NuG: NuGEN Ovation®, SMTer: SMARTer® Stranded; Tru4: TruSeq using 4 ng of RNA; Tru70: TruSeq using 70 ng of RNA. SMTseq4: SMART-Seq® v4 using 4 ng of RNA; SMTseq0.25: SMART-Seq® v4 using 250 pg of RNA. Yellow and orange: SMTseq samples; Red: SMTer samples; Black: NEB samples; Blue: NuGEN samples; Green and grey: TruSeq samples.. Color scale: Spearman correlation coefficients

In order to assess the similarity of expression profiles being generated by different library preparation kits, we applied Spearman correlation coefficients as a measure of similarity. The Spearman coefficient was calculated based on the rank of the CPM value as opposed to using the absolute values. This was done to accommodate the difference in CPM values due to differences in duplication rates observed among the kits (Fig. 5). The correlation coefficient for input samples ranged from 0.5 to 0.9, where SMARTseq profiles were better correlated with TruSeq70 than others (Spearman correlation coefficient ≥ 0.9). SMARTer samples had the lowest correlation (0.5) with the control library TruSeq70 (Fig. 5 and See Additional file 1: Table S4). Overall, as expected, input profiles are less correlated to corresponding IP profiles (See Additional file 1: Table S4). When we compared input samples with corresponding IP samples for each individual kit respectively, all input samples were clustered separately from IP samples except for the SMARTer samples (Additional file 4**:** Figure S3).

Although two different amounts of RNA were used for the TruSeq library kit, TruSeq 4 ng samples were well correlated with TruSeq 70 ng samples (Spearman correlation coefficient was 0.96 ± 0.002 for input; 0.946 ± 0.01 for IP). Similarly, the SMARTseq samples with 0.25 ng and 4 ng were highly correlated (Spearman correlation coefficient was 0.95 ± 0.004 for input and 0.95 ± 0.005 for IP) (See Additional file 1: Table S4).

### Transcript enrichment is better represented than transcript depletion in the IP samples

**Fig. 6:**
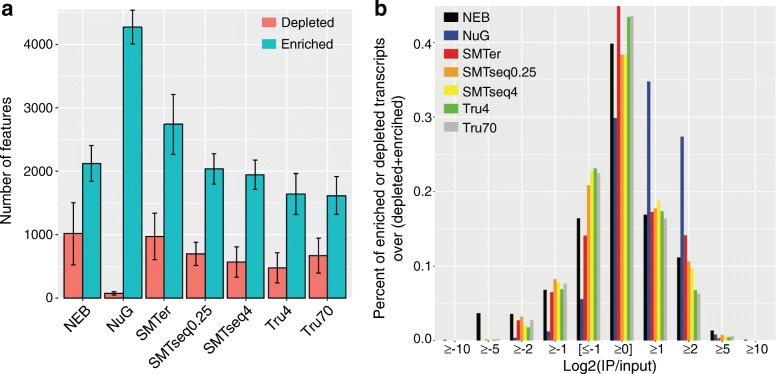
Descriptive characteristic of enrichment or depletion profiles as generated by the different library preparation kits. Genes which have at least 20 raw reads in the input samples and a ratio of IP/Input ≥2 or Input/IP ≥2 were used to generate the plots. **a** Total number of transcripts enriched or depleted. **b** Percentage of enriched or depleted transcripts grouped into different bins. X-axis: log2(IP/input), Y-axis: percentage of genes in each bin over whole population. NEB: NEBNext® Ultra™, NuG: NuGEN Ovation®, SMTer: SMARTer® Stranded; Tru4: TruSeq using 4 ng of RNA; Tru70: TruSeq using 70 ng of RNA. SMTseq4: SMART-Seq® v4 using 4 ng of RNA; SMTseq0.25: SMART-Seq® v4 using 250 pg of RNA. Yellow and orange: SMTseq samples; Red: SMTer samples; Black: NEB samples; Blue: NuGEN samples; Green and grey: TruSeq samples

**Fig. 7:**
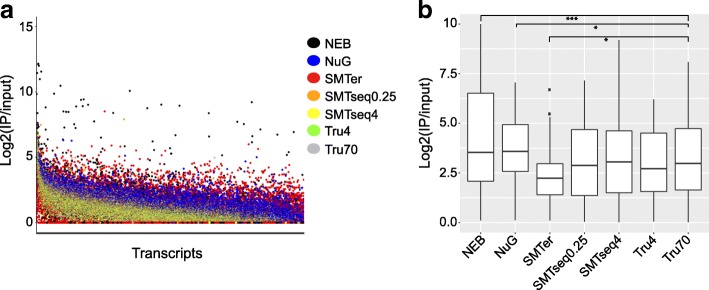
Enrichment profiles and top 50 enriched transcripts. **a** Enrichment factor of transcripts are sorted in decreasing order based on log2 (IP/input). X-axis:transcripts, Y-axis:log2 value of enrichment (IP/Input). **b** Boxplot of top 50 enriched transcripts. NEB: NEBNext® Ultra™, NuG: NuGEN Ovation®, SMTer: SMARTer® Stranded; Tru4: TruSeq using 4 ng of RNA; Tru70: TruSeq using 70 ng of RNA. SMTseq4: SMART-Seq® v4 using 4 ng of RNA; SMTseq0.25: SMART-Seq® v4 using 250 pg of RNA. Yellow and orange: SMTseq samples; Red: SMTer samples; Black: NEB samples; Blue: NuGEN samples; Green and grey: TruSeq samples

We evaluated the robustness of different kits for detecting enrichment (IP/input RNA > 1) or depletion (IP/input RNA < 1) of transcripts in the translatome (IP samples) compared to the transcriptome (input samples). Features with raw read counts ≥20 in input samples and with an enrichment or depletion factor ≥ 2 were included as enriched (IP/Input ≥2) or depleted transcripts (IP/Input ≤0.5). Of note, more transcripts were enriched than depleted (Fig. 6a and Additional file 5: Figure S4). NuGEN produced the greatest number of enriched transcripts (mean 4270) and smallest number of depleted transcripts (mean 74) as compared with other kits (Fig. 6b). Among the enriched transcripts from NuGEN, 60% were enriched less than 4-fold whereas only 25% of transcripts prepared by other kits were enriched less than 4-fold (Fig. 6b). NEB samples had the highest percentage of enriched/depleted transcripts (log2 (IP/INPUT) > 5 or log2(INPUT/IP) > 5) when compared to samples obtained from the other kits (Fig. 6b, Fig. 7a). Conversely, the enrichment profile of the SMARTer samples showed fewer enriched or depleted transcripts compared with the rest of the samples. Indeed, when plotting for the top 50 enriched transcripts (Fig. 7b), the median enrichment value for the SMARTer profile was significantly lower than other profiles (*p* < 0.05).

We also compared the number of transcripts being enriched or depleted across samples (Additional file 6: Figure S5). NuGEN had the highest number of uniquely enriched transcripts that were detected (accounting for 25% of its total enriched transcripts, 95% of which are protein-coding genes). TruSeq4 and TruSeq70 had around 5% uniquely enriched transcripts (Additional file 6: Figure S5a,b).

**Fig. 8:**
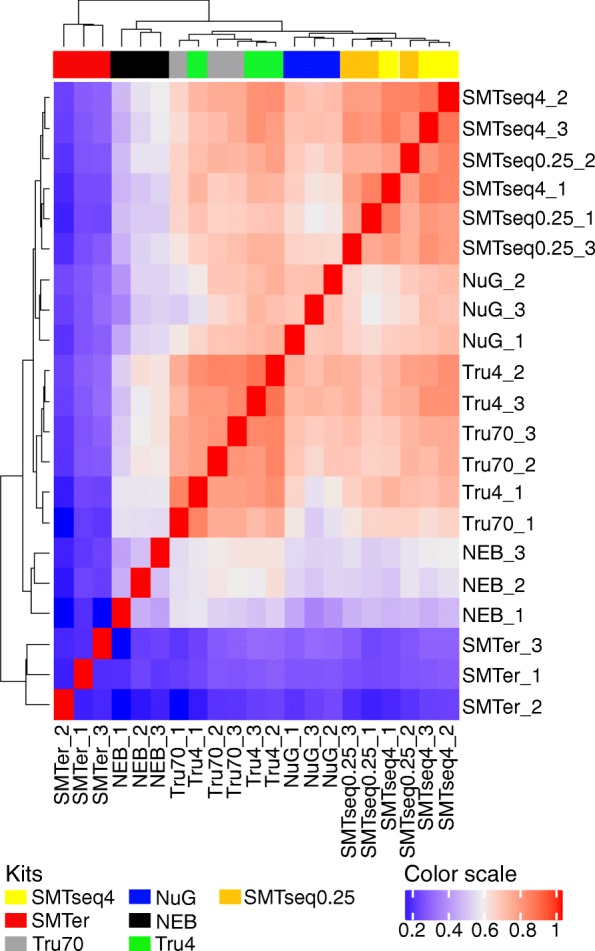
Hierarchical clustering based on the rank of IP/input value. Dendrogram represents Spearman correlation coefficients between pairs of samples. NEB: NEBNext® Ultra™, NuG: NuGEN Ovation®, SMTer: SMARTer® Stranded; Tru4: TruSeq using 4 ng of RNA; Tru70: TruSeq using 70 ng of RNA. SMTseq4: SMART-Seq® v4 using 4 ng of RNA; SMTseq0.25: SMART-Seq® v4 using 250 pg of RNA. Yellow and orange: SMTseq samples; Red: SMTer samples; Black: NEB samples; Blue: NuGEN samples; Green and grey: TruSeq samples

We also clustered all the transcripts based on the rank of enrichment factor or depletion factor greater than 2 in at least one sample (Fig. 8). As expected, the profiles for TruSeq4 and TruSeq70 were most similar to each other (Spearman correlation coefficient > 0.7). The same is true for SMARTseq4 and SMARTseq0.25. On the other hand, the enrichment/depletion profile for SMARTer was the least similar to the other profiles (Spearman correlation coeffienct < 0.2).

### Transcript-length dependent enrichment/depletion

**Fig. 9:**
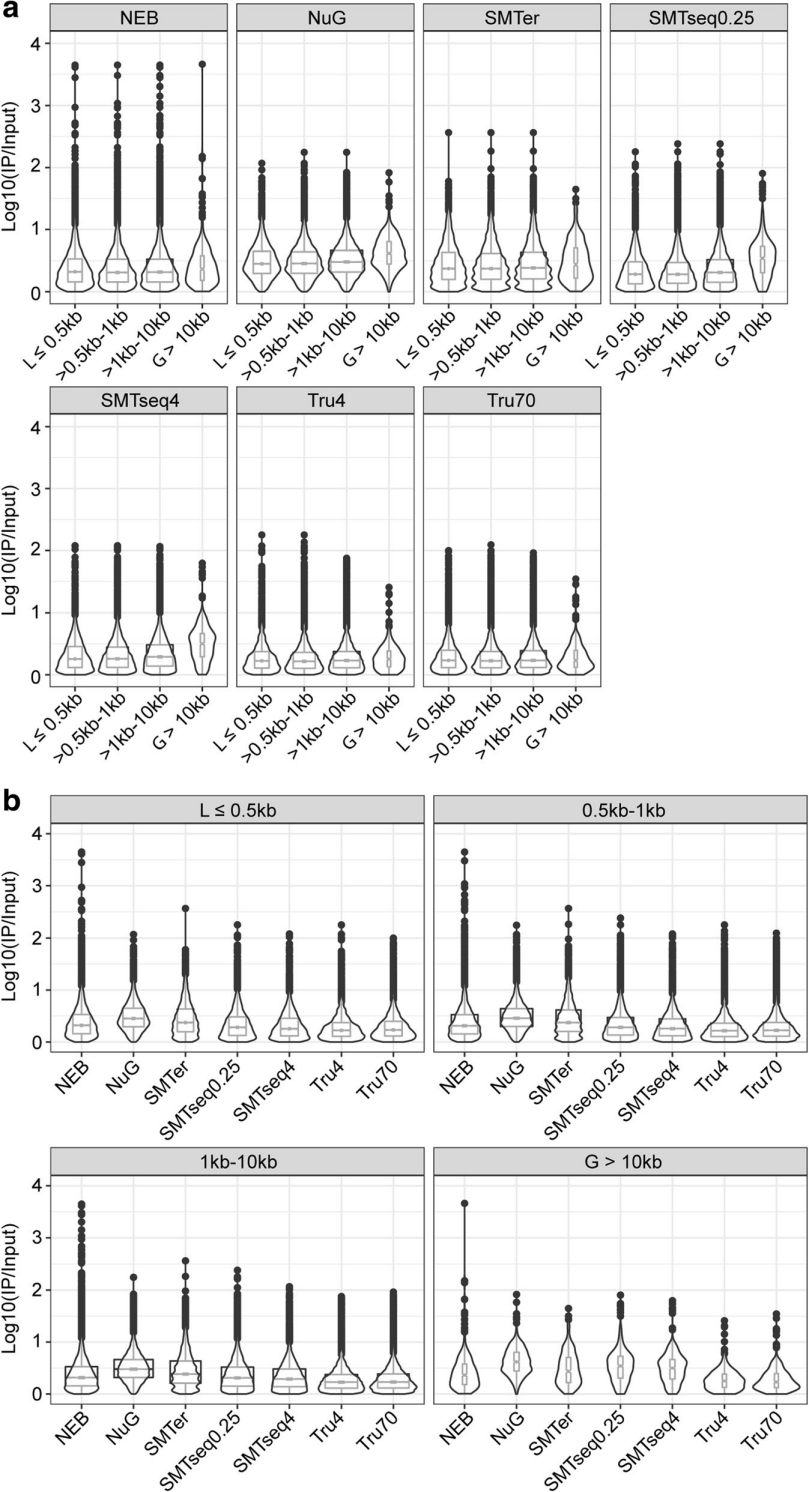
Boxplot and violin plots of enriched features over different transcript lengths. **a** Boxplots of enrichment factor of different transcripts within each kit. **b** Boxplots of enrichment factor of different kits at each transcript length bin. The notch represents the median ± 1.58IQR/√n. The width of boxplot is proportion to sample size of each group

We examined whether the enrichment or depletion effects observed in the translatome were affected by the transcript length. Based on size distribution of the enriched/depleted transcripts (the majority being between 0.5 and 10 kb, Additional file 7: Figure S6), we grouped the transcripts into four bins (≤0.5 kb, 0.5–1 kb, 1 kb–10 kb, and > 10 kb) (Fig. 9). The median enrichment for transcripts was relatively higher in the longer transcript (> 10 kb) except in TruSeq70 samples (Fig. 9a). Within each transcript length bin, the median enrichment effect from NuGEN and SMARTer samples was much higher than TruSeq70 samples for transcripts less than 10 kb (Fig. 9b). For longer transcripts (> 10 kb), NEB, NuGEN, SMARTer and SMARTseq samples had a median enrichment that is much higher than those of TruSeq70 (Fig. 9b). Additionally, the enrichment effect for NEB samples distributed wider than all the other samples (Fig. 9).

**Fig. 10:**
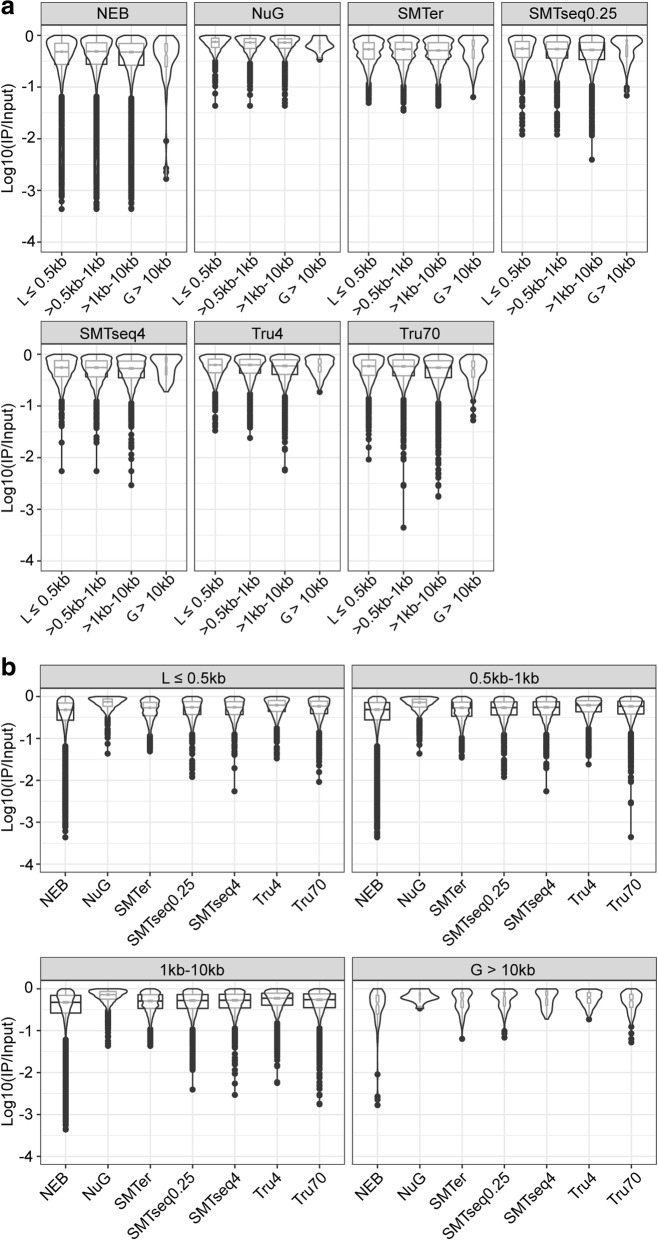
Boxplot and violin plots of depletion features over different transcript lengths. **a** Boxplots of depletion factor of different transcripts within each kit. **b** Boxplots of depletion factor of different kits at each transcript length bin. The notch represents the median ± 1.58IQR/√n. The width of boxplot is proportion to sample size of each group

A similar trend was also observed in depleted transcripts. Across all transcript lengths, the range of depletion effect for NuGEN and SMARTer samples was less than for other samples (Fig. 10a). For NEB samples, the depletion effect distribution was wider than all the other samples (Fig. 10b). For longer transcripts (> 10 kb), NEB, NuGEN, SMARTer and SMARTseq samples showed fewer depletion effects than those from TruSeq70 (Fig. 10b).

## Discussion

In this study, we compared five library-preparation kits for RNA-seq, using low-quantity input RNA or RiboTag IP RNA, by applying a comprehensive set of quality measures. One of the major differences among library preparation kits was whether oligo (dT) is used to select mRNA. Among the kits tested, the NEBNext® Ultra™, the Illumina TruSeq® and the TaKaRa SMART-Seq® v4 Ultra® use oligo-dT primers to select for polyA mRNA. Conversely, the TaKaRa SMARTer® kit depends on locked nucleic acid (LNA) technology and random primers to capture both products with classical long polyA(+) and those with short poly(A) tails or polyA(−) transcripts and employs a ribosomal depletion step. Although the NuGEN Ovation® V2 kit uses a combination of semi-random hexamers and a poly-dT chimeric primer for 1st strand cDNA amplification in an effort to mitigate bias, 3′ end bias was still observed. Shanker, et al.(2015), also observed 3′ end bias using the NuGEN Ovation V2 kit with low input samples [22]. Interesingly, we observed greater 3′ end bias in IP samples (except for SMARTer prepared samples) than in the input samples, possibly suggesting some degradation of the RNA.

A higher percentage of reads mapped to the intronic and intergenic region in the samples derived from the TaKaRa SMARTer® kit in comparison to samples derived from the other five kits. Adiconis, et al., 2013 [23], also found a similar difference by comparing the SMARTer® kit to the TruSeq® kit. In our study, among the top-100 highly expressed transcripts in SMARTer samples, 30% were miRNA, lincRNA and rRNA. It is known that the source of miRNA or lincRNA is mainly from intergenic or intronic region, and that certain ribosomal RNAs generated by RNA polymerase I and III are without polyA tails. Therefore, we propose that the SMARTer® kit may be useful for studies which aim to focus on poly(A) negative transcripts or transcripts derived from non-exon-coding regions.

We observed lower duplication rates for the SMART-Seq® v4 Ultra (SMARTseq) and NuGEN prepared samples as compared to samples prepared with the other kits. A possible explanation to this observed lower duplication rate may relate to the protocols of these two kits. The mRNA is pre-amplifed to cDNA, before fragmentation, making the duplication rate resulting from the amplification harder to identify based on mapping position. Conversely, in other methods including the TruSeq kit, mRNA is fragmented first and the amplification only happens during the library construction step, making it easier to identify duplication based on the mapping position. For this study, the comparisons among different kits were achieved by using the rank-based method without removal of duplicate reads. Parekh et al., also showed that removal of duplicates improved neither accuracy nor precision and can actually worsen the power and the False Discovery Rate (FDR) for differential gene expression [33].

While Combs et al., (2015) [34] reported the use of the TruSeq kit with 100 ng of RNA, our modification of the TruSeq protocol provides the possibility to use the kit with RNA amounts as low as 4 ng. Indeed, our study, for the first time, shows that with protocol modifications, TruSeq with 4 ng of RNA performs similarly to the TruSeq with 70 ng of RNA with respect to the number of genes being captured and overall profile composition.

Comparing translatome (IP samples) against corresponding transcriptome (input samples), we find a relative higher intronic percentage in the translatome profiles, which might indicate that some non-mature RNA are precipitated during the IP process. Overall, we detected more enriched transcripts than depleted transcripts in the IP samples. Roh et al., report a similar result although the fold-change was greater in the depleted genes than the enriched genes [35]. This difference may result from the different tissues being used, and more specifically the percent of cells that express the tagged ribosomes. Among enriched transcripts, we observed an enrichment effect bias toward longer transcripts (> 10 kb) (Fig. 9a). This may relate to the nature of RiboTag IP since it is a method to detect polysome profiles during translation [16]. It is possible that the higher number of ribosomes on longer transcripts leads to a higher enrichment. In addition, the greater enrichment effect of longer transcript is slightly higer for samples prepared by SMARTer and SMARTseq kits. This may be related to the template-switch oligonucleotide with one locked nucleic acid (LNA) technique applied in these kits, which is aimed to improve the hybridization between the template-switch oligonucleotide and the cDNA product [36, 37], increasing full coverage for longer transcripts.

## Conclusion

Amongst the kits and library prep protocols analyzed in this manuscript, SMART-Seq v4 and TruSeq offer the best sequencing results for libray preparation from smaller amounts of RNA as starting material. Indeed, the overall profile of 250 pg/4 ng samples from SMART-Seq v4 was similar to the TruSeq 70, here used as a gold-standard control. SMARTer Stranded Total RNA-Seq Kit might be a good choice to study both polyA(+) mRNA and non-polyA mRNA, especially non-coding RNAs. Since there is a coverage bias towards 3′ for IP samples and more enrichment for longer transcripts, correction should be included during comparison among samples, for example, using the bias correction function in Cufflink [38]. Finally, IP RNA from RiboTag samples is likely to include a higher rate of immature RNAs, given the observed increase in intronic sequences in the IP samples across all library prep approaches. Overall, we were able to observe both enriched and depleted transcripts of translatome profiles using all kits. Greater enrichment effects were detected than depletion, however this may be related to the percent of tagged ribosomes in the tissue and therefore tissue and Cre-driver specific. In summary, by considering the eveness of coverage, number of detected features, low CPM of non-coding genes, and similar enrichment profiles comparing to standard TruSeq70 prepared samples, the SMARTseq and NEB kits performed the best in comparison to the other kits tested. However, the SMARTseq kit had a lower duplication rate and allows reactions to start with as little as 250 pg, significantly decreasing the necessary amount of starting material. In addition, the modified TruSeq4 protocol provides good results based on the relative high number of detected features, low CPM of non-coding genes, and similarity of the enrichment profile to the standard TruSeq70.

## Additional files

Additional file 1: **Table S1.** Library preparation performed in this study. Table S2 Gene list with RPKM greater than 10,000. Table S3 Median of genes from unique and shared regions of Venn diagram. Table S4 Spearman correlation coefficients between different profiles. (XLSX 28 kb)

Additional file 2: **Figure S1.** Cre recombination in liver cells expressing Gfi1. (a) Cryosection of liver from a cross between a Gfi1-Cre mouse and a TdTomato reporter mouse (Ai14) stained with DAPI and phalloidin. TdTomato is found in a subset of cells in the liver that is likely consistent with Kupffer or endothelial cells. (b) Enrichment of *Gfi1* transcripts in the IP samples as compared to the input samples was assessed by reverse-transcription followed by real time PCR. (PDF 2947 kb)

Additional file 3: **Figure S2.** Duplication rate of each library. X-axis: duplication rate, Y-axis: log10 of reads at different duplication rates. (PDF 622 kb)

Additional file 4: **Figure S3.** Hierarchical clustering of expression levels, based on the rank of the count of exon per million mapped reads (CPM). Dendrograms represent Spearman correlation coefficients between pairs of samples that is 3 replicates for input and 3 replicates for IP. (PDF 233 kb)

Additional file 5: **Figure S4.** Bland–Altman plot (MA plot) of translatome (IP samples) and transcriptome (input samples) profiles for each kit. The red lines represent the boundary cutoff [− 1,1]. Dots above or below the red line represent the enriched or depleted transcripts. (JPG 587 kb)

Additional file 6: **Figure S5.** Venn diagram of enriched/depleted transcripts (CPM ≥ 20 in at least one replicate, mean ratio of enrichement/depletion of the three replicates). The mean ratio IP/input is ≥2 or input/IP is ≥2. (PDF 1063 kb)

Additional file 7: **Figure S6.** Histogram of length distribution for enriched or depleted transcripts. (PDF 353 kb)

## Abbreviations

NEB: NEBNext® Ultra™ Directional RNA Library Prep Kit for Illumina with 4 ng of RNA
NuGEN: NuGEN Ovation® RNA-Seq System V2 with 4 ng of RNA
SMARTer: TaKaRa SMARTer® Stranded Total RNA-Seq Kit-Pico Input Mammalian with 4 ng of RNA
SMARTseq4 and SMARTseq0.25: TaKaRa SMART-Seq® v4 Ultra® Low Input RNA Kit for Sequencing with 4 ng and 0.25 ng of RNA
TruSeq4 and TruSeq70: Illumina TruSeq RNA Library Prep Kit v2 with 4 ng and 70 ng of RNA
