# Supplementary material for: A comparative analysis of library prep approaches for sequencing low input translatome samples
Source: BMC Genomics. 2018 Sep 21;19:696. doi: 10.1186/s12864-018-5066-2 (PMC6151020; doi:10.1186/s12864-018-5066-2)
Supplement: Supplementary file 2 — Figure S1. Cre recombination in liver cells expressing Gfi1. (a) Cryosection of liver from a cross between a Gfi1-Cre mouse and a TdTomato reporter mouse (Ai14) stained with DAPI and phalloidin. TdTomato is found in a subset of cells in the liver that is likely consistent with Kupffer or endothelial cells. (b) Enrichment of Gfi1 transcripts in the IP samples as compared to the input samples was assessed by reverse-transcription followed by real time PCR. (PDF 2947 kb) [file 12864_2018_5066_MOESM2_ESM.pdf]

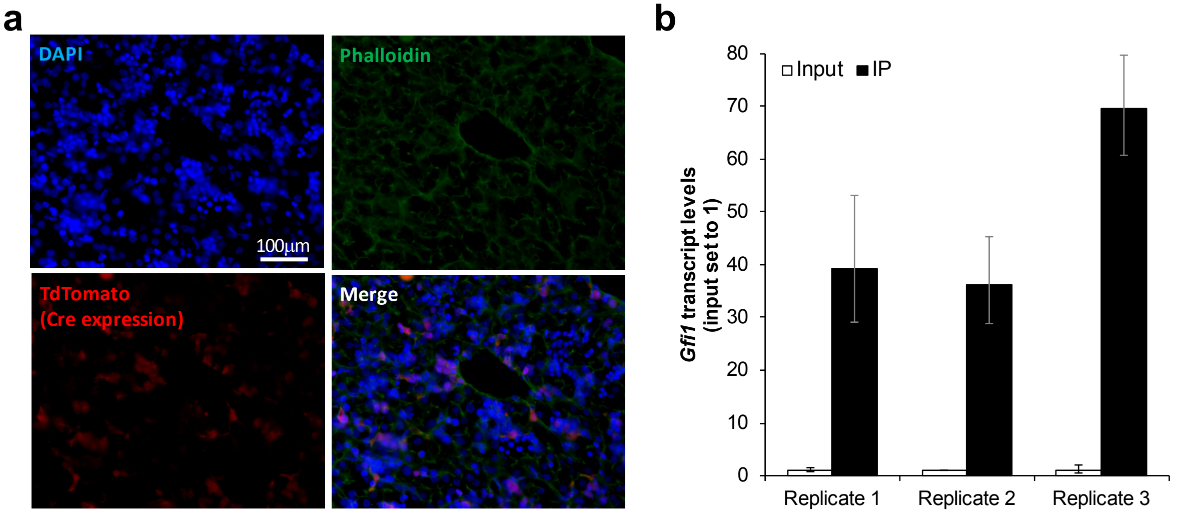

Fig. *S*1 Cre recombination in liver cells expressing *Gfi1*.
(a) Cryosection of liver from a cross between a Gfi1-Cre mouse and a TdTomato reporter mouse (Ai14) stained with DAPI and phalloidin. TdTomato is found in a subset of cells in the liver that is likely consistent with Kupffer or endothelial cells. (b) Enrichment of Gfi1 transcripts in the IP samples as compared to the input samples was assessed by reverse-transcription followed by real time PCR.
