# Supplementary figures and images for: A comparative analysis of library prep approaches for sequencing low input translatome samples
Source: BMC Genomics. 2018 Sep 21;19:696. doi: 10.1186/s12864-018-5066-2 (PMC6151020; doi:10.1186/s12864-018-5066-2)

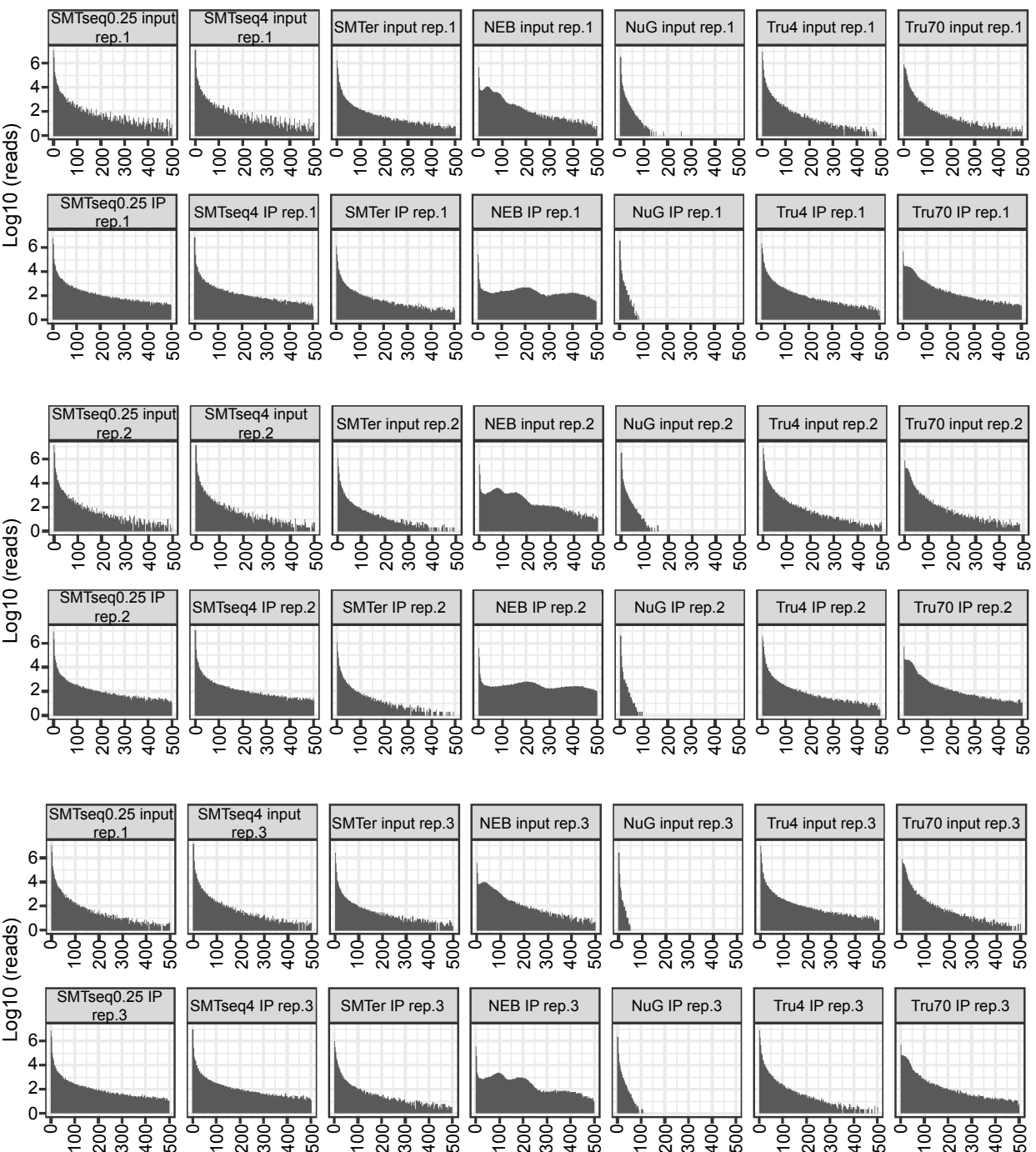

Fig. S2 Duplication rate of each library

Supplement: Supplementary file 3 — Figure S2. Duplication rate of each library. X-axis: duplication rate, Y-axis: log10 of reads at different duplication rates. (PDF 622 kb) [file 12864_2018_5066_MOESM3_ESM.pdf]

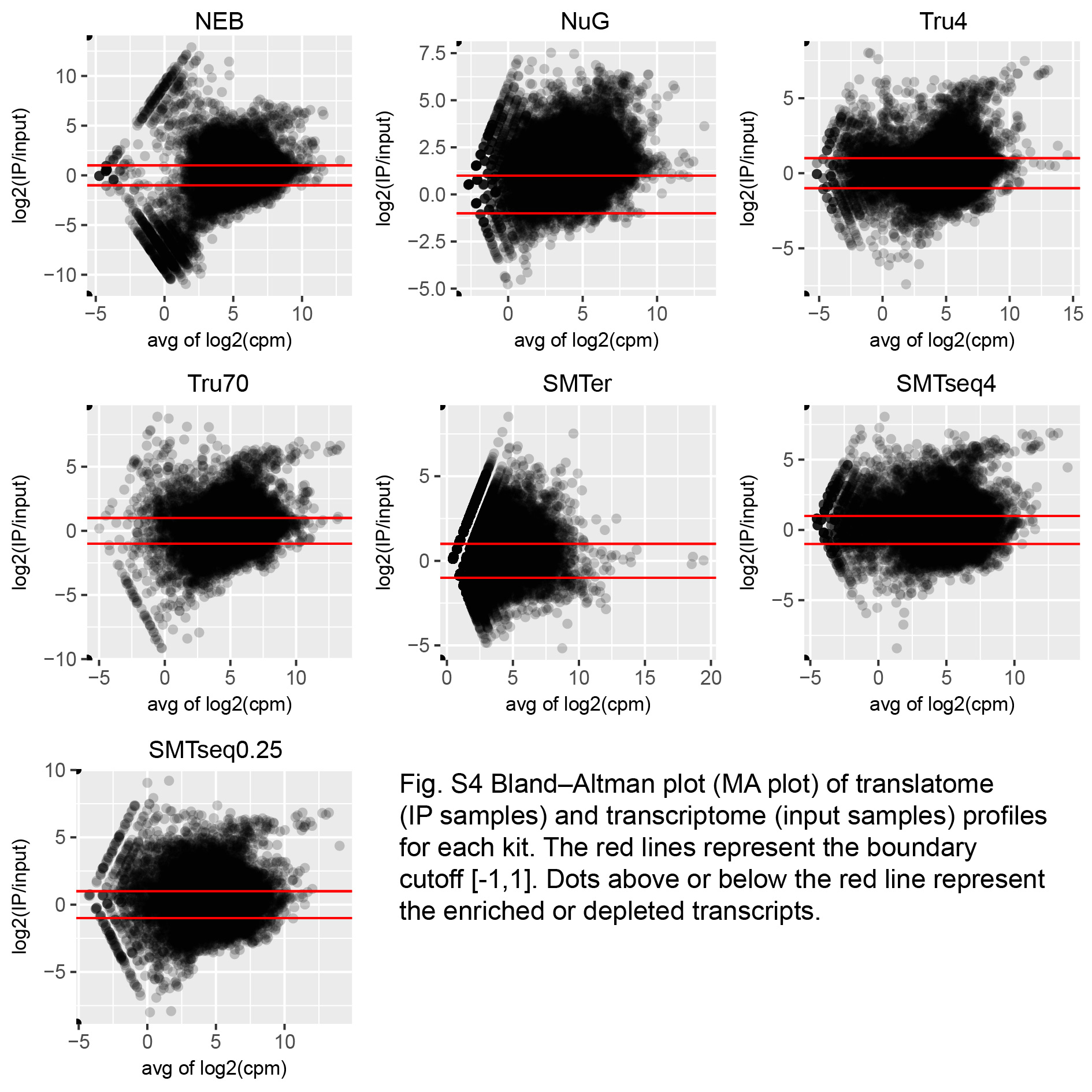

Supplement: Supplementary file 5 — Figure S4. Bland–Altman plot (MA plot) of translatome (IP samples) and transcriptome (input samples) profiles for each kit. The red lines represent the boundary cutoff [− 1,1]. Dots above or below the red line represent the enriched or depleted transcripts. (JPG 587 kb) [file 12864_2018_5066_MOESM5_ESM.jpg]

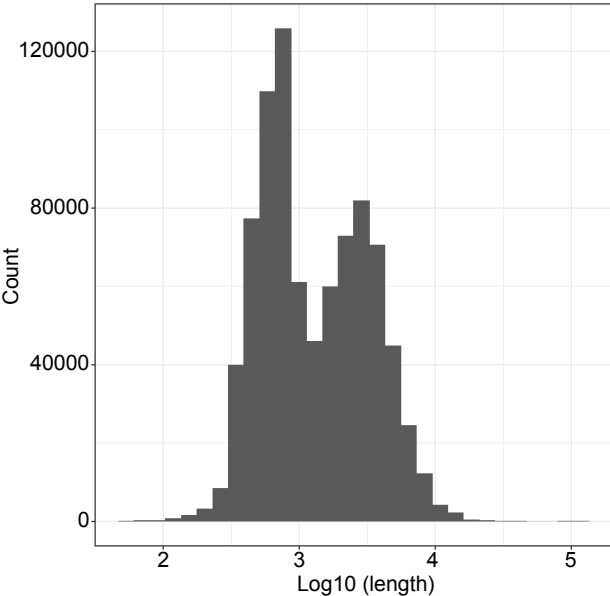

Fig. S6 Histogram of length distribution for enriched or depleted transcripts

Supplement: Supplementary file 7 — Figure S6. Histogram of length distribution for enriched or depleted transcripts. (PDF 353 kb) [file 12864_2018_5066_MOESM7_ESM.pdf]
