# Supplementary material for: A comparative analysis of library prep approaches for sequencing low input translatome samples
Source: BMC Genomics. 2018 Sep 21;19:696. doi: 10.1186/s12864-018-5066-2 (PMC6151020; doi:10.1186/s12864-018-5066-2)
Supplement: Supplementary file 4 — Figure S3. Hierarchical clustering of expression levels, based on the rank of the count of exon per million mapped reads (CPM). Dendrograms represent Spearman correlation coefficients between pairs of samples that is 3 replicates for input and 3 replicates for IP. (PDF 233 kb) [file 12864_2018_5066_MOESM4_ESM.pdf]

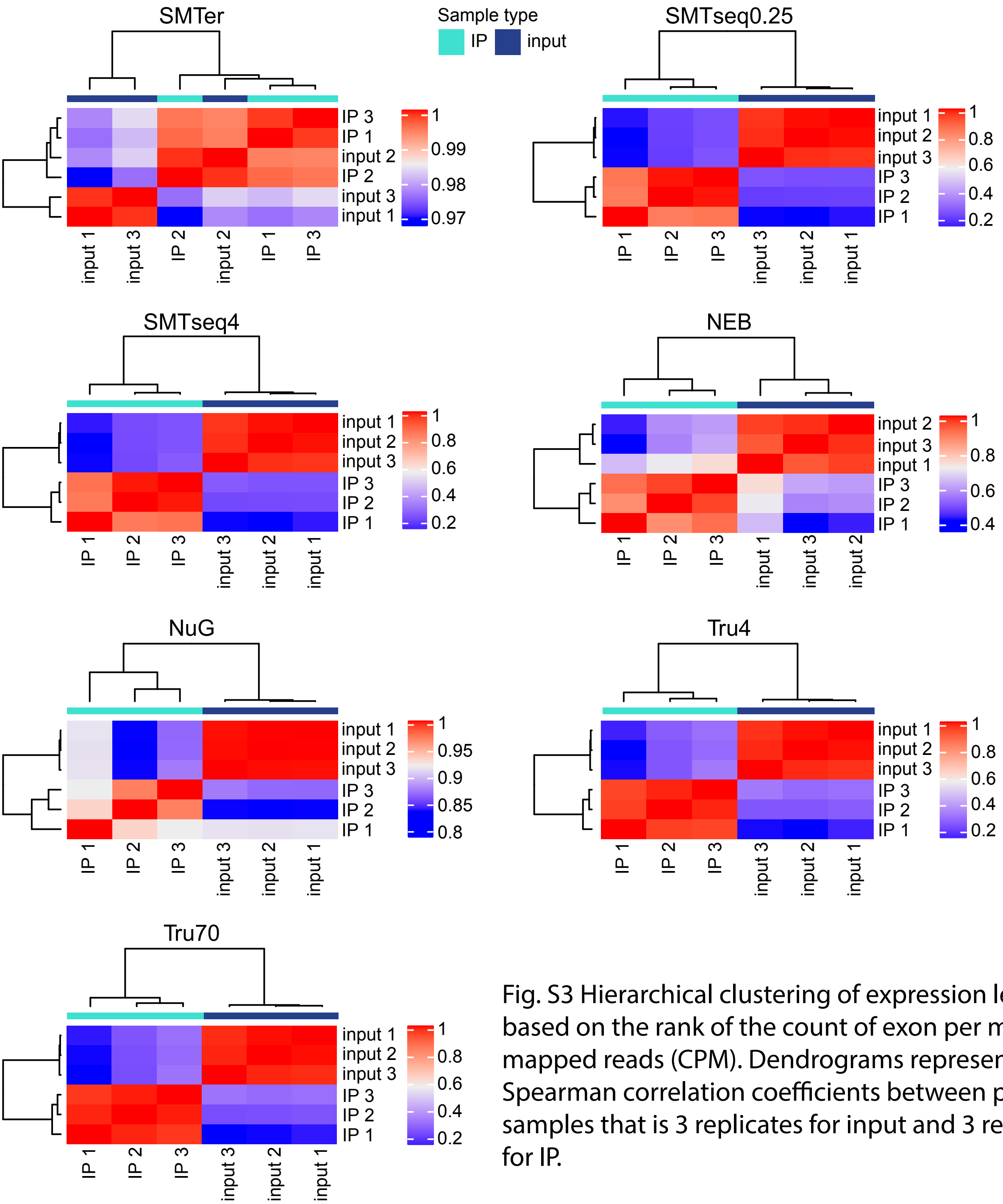

Fig. S3 Hierarchical clustering of expression levels, based on the rank of the count of exon per million mapped reads (CPM). Dendrograms represent Spearman correlation coefficients between pairs of samples that is 3 replicates for input and 3 replicates for IP.
