# Supplementary material for: A comparative analysis of library prep approaches for sequencing low input translatome samples
Source: BMC Genomics. 2018 Sep 21;19:696. doi: 10.1186/s12864-018-5066-2 (PMC6151020; doi:10.1186/s12864-018-5066-2)
Supplement: Supplementary file 6 — Figure S5. Venn diagram of enriched/depleted transcripts (CPM ≥ 20 in at least one replicate, mean ratio of enrichement/depletion of the three replicates). The mean ratio IP/input is ≥2 or input/IP is ≥2. (PDF 1063 kb) [file 12864_2018_5066_MOESM6_ESM.pdf]

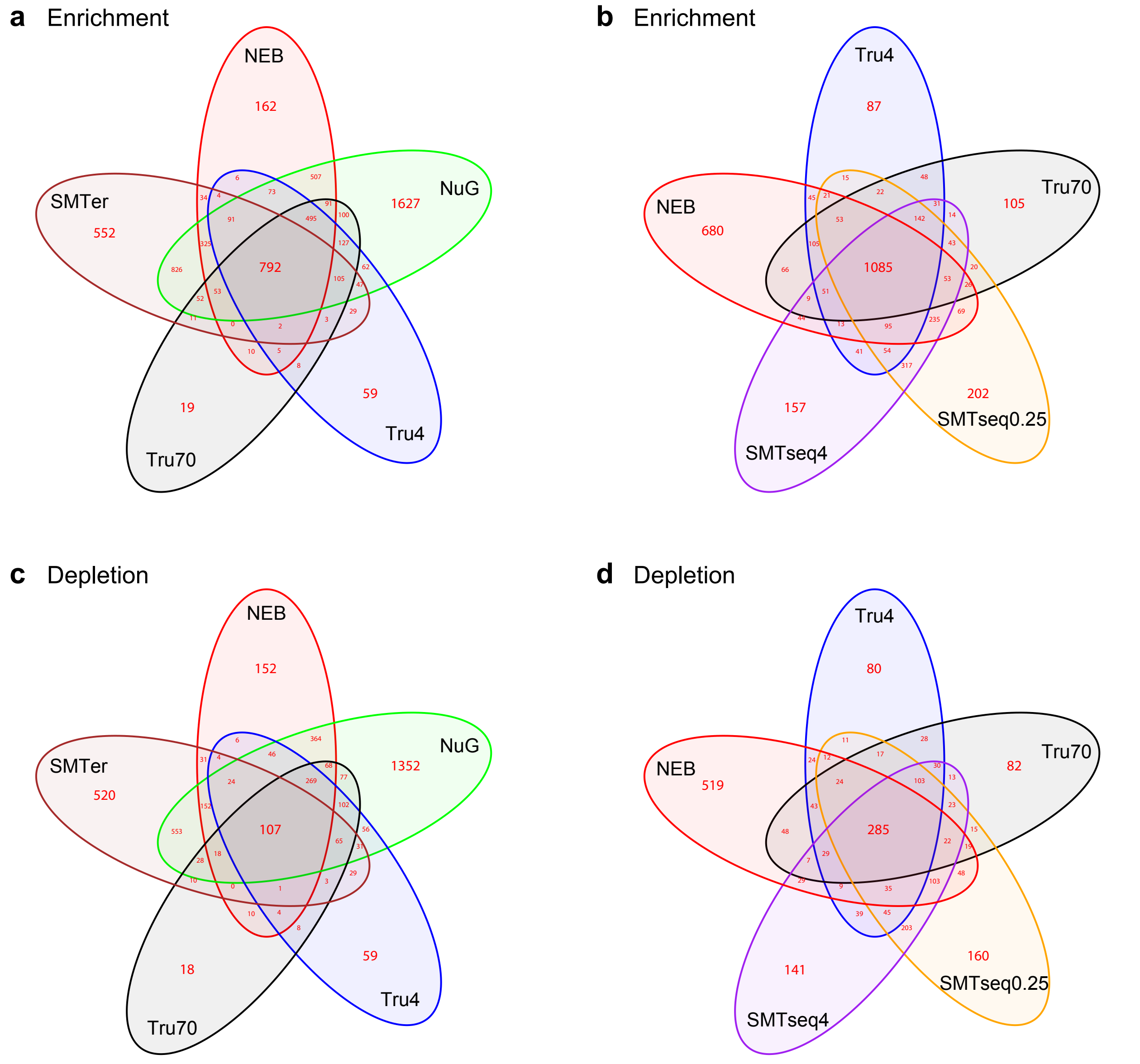

Fig. S5 Venn diagram of enriched/depleted transcripts (CPM ≥ 20 in at least one replicate, mean ratio of enrichement/depletion of the three replicates). The mean ratio IP/input is ≥2 or input/IP is ≥2.
